# 探索提高肺癌脑膜转移脑脊液细胞学病理检测阳性率的方法及影响因素

**DOI:** 10.3779/j.issn.1009-3419.2022.102.42

**Published:** 2022-11-20

**Authors:** 乃升 高, 冲 滕, 承娟 范, 涛 信

**Affiliations:** 150081 哈尔滨，哈尔滨医科大学附属第二医院肿瘤内科 Department of Oncology, The Second Affiliated Hospital of Harbin Medical University, Harbin 150081, China

**Keywords:** 脑膜转移癌, 肺肿瘤, 脑脊液细胞学, 脑脊液生化检查, 诊断, Leptomeningeal metastases, Lung neoplasms, Cerebrospinal fluid cytology, Biochemical examination of cerebrospinal fluid, Diagnosis

## Abstract

**背景与目的:**

脑膜转移癌（leptomeningeal metastases, LM）是晚期非小细胞肺癌（non-small cell lung cancer, NSCLC）中进展迅速、预后差的灾难性转移性事件。NSCLC患者的LM发病率约为3%-5%，伴有表皮生长因子受体（epidermal growth factor receptor, *EGFR*）突变患者的发病率高达9.4%。脑脊液细胞病理学是诊断LM的金标准，但是常规细胞病理学阳性诊断率一般不超过50%，导致LM诊断延迟、治疗延误。脑脊液样本固定处理是影响细胞学阳性检出的一个重要因素，如何提高脑脊液细胞病理学阳性检出率是目前临床研究热点。

**方法:**

收集2019年6月-2021年11月于哈尔滨医科大学附属第二医院肿瘤内科二病房收治的105例依据临床症状和影像学阳性表现诊断为LM的病例进行回顾性分析。根据不同送检方式分为应用存有TIB细胞保存液试剂盒（实验组）和常规应用腰穿包内无菌塑料试管（对照组）收集并送检脑脊液的标本，探索不同固定方式对脑脊液细胞病理学阳性率的影响。同时在不分组条件下送检脑脊液进行生化检测（葡萄糖、总蛋白），建立*Logistic*回归分析及受试者工作特征曲线（receiver operating characteristic curve, ROC）评估对肺癌LM患者的辅助诊断价值。动态监测培美曲塞鞘内注射治疗后脑脊液生化（葡萄糖、总蛋白）指标与细胞学变化，研究生化指标对临床疗效的评估价值。

**结果:**

实验组中，42例（80.77%）患者首次脑脊液细胞学检查结果为阳性，10例（19.23%）患者结果为阴性，对照组中24例（45.28%）患者首次脑脊液细胞学检查结果为阳性，29例（54.72%）患者首次检查结果为阴性，两组差异有统计学意义（*P* < 0.001）。首次行脑脊液生化检测的患者*Logistic*回归分析结果显示，脑脊液葡萄糖 < 2.5 mmol/L发生脑脊液细胞病理学阳性风险是脑脊液葡萄糖≥2.5 mmol/L的2.456倍（OR=2.456, *P* < 0.05），脑脊液总蛋白≥430 mg/L发生脑脊液细胞病理学阳性风险是脑脊液总蛋白 < 430 mg/L的2.647倍（OR=2.647, *P* > 0.05）。ROC曲线显示，脑脊液中葡萄糖灵敏度为76.9%，特异度为54.5%，Youden指数为0.315，曲线下面积（area under the curve, AUC）为0.620；脑脊液中总蛋白灵敏度为44.4%，特异度为90.6%，Youden指数为0.350，AUC为0.671。分别对73例、50例行2个周期培美曲塞鞘内注射治疗后，并有完整的脑脊液细胞学和脑脊液生化（葡萄糖、总蛋白）检测患者进行动态监测，脑脊液细胞学转阴率均逐渐升高。脑脊液葡萄糖水平治疗2个周期后较首次升高，差异有统计学意义（*P* < 0.001）。

**结论:**

具有临床症状和影像学阳性的患者脑脊液样本离体后应用存有细胞保存液试剂盒立即固定，可以显著提高脑脊液细胞学阳性检出率。脑脊液生化检测尤其是葡萄糖含量的降低对LM具有辅助诊断价值，连续动态监测脑脊液生化及细胞学可以评估治疗效果并且具有预测的作用。

## Abstract (en)

**Background and objective:**

In advanced non-small cell lung cancer (NSCLC), leptomeningeal metastases (LM) is a common consequence with rapid progression and a poor prognosis. LM affects roughly 3% to 5% of NSCLC patients, and it affects as many as 9.4% of individuals with epidermal growth factor receptor (*EGFR*) mutations. Cerebrospinal fluid cytology is the gold standard for diagnosing LM, while conventional cytopathology has a positive detection rate of less than 50%, resulting in a delay in diagnosis and treatment of LM. The fixation treatment of cerebrospinal fluid samples has a significant impact on the positive cytology detection rate, and how to improve the positive cytopathology detection rate of cerebrospinal fluid is a hot topic in clinical research.

**Methods:**

From June 2019 to November 2021, 105 cases diagnosed with LM based on clinical symptoms and positive imaging were collected and retrospectively evaluated in the second ward of the Department of Oncology of The Second Affiliated Hospital of Harbin Medical University. The effect of different fixation methods on the positive rate of cerebrospinal fluid cytopathology was investigated, and specimens of cerebrospinal fluid were collected and sent for examination using different delivery methods, including the application of the TIB cell preservation solution kit (experimental group) and the routine application of sterile plastic tubes in lumbar puncture bags (control group). Biochemical assays (glucose and total protein) were performed on the cerebrospinal fluid fluid, and *Logistic* regression analysis and receiver operating characteristic (ROC) curve were used to evaluate the supplementary diagnostic value for LM patients with lung cancer. The relevance of chemical indexes in the assessment of therapeutic efficacy was examined, and biochemical (glucose, total protein) indices and cytological changes in cerebrospinal fluid fluid after pemetrexed intrathecal injection therapy were dynamically monitored.

**Results:**

In the control group, 24 (45.28%) patients were positive for the first time, while 42 (80.77%) patients were positive for the first time and 10 (19.23%) patients were negative for the first time in the experimental group. Significant differences existed between the two groups (*P* < 0.001). The results of *Logistic* regression analysis of patients with the first cerebrospinal fluid biochemical test showed that the risk of positive cerebrospinal fluid biochemical pathology with less than 2.5 mmol/L was 2.456 times greater than 2.5 mmol/L of cerebrospinal fluid glucose (OR=2.456, *P* < 0.05), and total cerebrospinal fluid biochemical protein greater than 430 mg/L was 2.647 times less than 430 mg/L (OR=2.647, *P* > 0.05). The ROC curve showed glucose sensitivity of 76.9% in cerebrospinal fluid, the specificity of 54.5%, Youden index of 0.315 and area under the curve (AUC) of 0.620, total protein sensitivity in cerebrospinal fluid of 44.4%, 90.6%, Youden index of 0.350 and AUC of 0.671. After 2 cycles of pemetrexed intrathecal treatment with complete cerebrospinal fluid cytology and cerebrospinal fluid biochemical (glucose, total protein) tests in 73 and 50 patients, respectively, the rate of cerebrospinal fluid cytology turning negative was gradually increased. Cerebrospinal fluid glucose levels increased after 2 cycles of treatment compared with the first time, with a statistically significant difference (*P* < 0.001).

**Conclusion:**

The use of a cell preservation solution kit to immediately fix cerebrospinal fluid samples following isolation in patients with clinical symptoms and positive imaging greatly enhances the rate of positive cerebrospinal fluid cytology detection. The effect of treatment can be assessed and predicted by continuous dynamic monitoring of cerebrospinal fluid biochemistry and cytology.

近年来，肺癌靶向治疗及免疫治疗进展迅速，肺癌患者生存期延长，但肺癌脑膜转移癌（leptomeningeal metastases, LM）发病率正在不断升高^[1, 2]^。因LM临床症状复杂，影像学表现不典型，最终只能依靠脑脊液细胞病理学进行确定诊断，所以临床诊断、治疗往往滞后于临床表现，进而影响了肺癌LM的治疗及预后^[3]^。脑脊液中检出肿瘤细胞是LM诊断的金标准，传统的脑脊液固定送检方式为普通塑料试管封闭后送检离心、液基涂片检查，其细胞病理学阳性率不高^[4]^，而脑脊液离体后蛋白会很快降解，这种保存方式是否为影响脑脊液阳性率的因素，脑脊液样本离体后立即固定是否会增加脑脊液细胞学阳性检出率，目前尚无此方面的研究。

鞘内化疗（intrathecal chemotherpy, ITC）可以通过腰椎穿刺等方法将抗肿瘤药物直接注入脑脊液中，被认为是治疗LM的可靠手段。培美曲塞作为非小细胞肺癌（non-small cell lung cancer, NSCLC）的一线治疗常用药物，本课题组综合临床及基础研究成果，于2018年注册并完成首个国际原创性单臂二期临床研究培美曲塞联合地塞米松鞘内注射治疗表皮生长因子受体（epidermal growth factor receptor, *EGFR*）突变肺癌多线酪氨酸激酶抑制剂（tyrosine kinase inhibitors, TKIs）治疗失败后LM安全性及疗效研究（注册号：ChiCTR1800016615）。该研究探索了培美曲塞鞘内注射的安全剂量，研究结果显示培美曲塞鞘内注射多线靶向治疗失败肺癌LM取得很好的疗效（客观有效率超过80%）中位总生存期（median overall survival, mOS）超过9个月，安全性也明显好于传统药物^[5]^，并将培美曲塞鞘内注射写入LM治疗指南^[6-9]^。在试验过程中我们发现采用传统固定方式细胞病理阳性率不足50%，是否因脑脊液固定方式的问题导致阳性检出率低成为我们的研究热点问题。2021年1月经与我院病理科沟通，临床工作中采用TIB细胞保存液试剂盒保存脑脊液的方式后，我们发现脑脊液细胞病理学阳性检出率显著提高，因此本试验回顾性分析通过不同脑脊液固定方式对脑脊液细胞学并配合脑脊液生化检查的研究，探索提高LM的诊断方法，及其在判断预后的临床应用价值，旨在建立一种简便、可靠、高效的系统立体诊断方法，为LM的机制研究诊断和治疗提供保障。

## 1 材料与方法

### 1.1 一般资料

收集2019年6月-2021年11月于哈尔滨医科大学附属第二医院肿瘤内科二病房收治的105例依据临床症状和影像学阳性表现诊断为肺腺癌脑膜转移，同时于我科接受培美曲塞鞘内化疗的患者。①既往明确肺腺癌病史的患者；②新近出现的临床症状/体征，如头痛、恶心、呕吐等精神神经症状；③典型的如脑膜强化等影像学表现；④充分知情同意告知；其中LM的诊断需同时满足条件①的前提下加上②、③或其中之一。排除标准：①合并严重全身感染、局部感染或严重的基础疾病；②存在意识障碍；③心肺功能差不能耐受后续治疗。

### 1.2 试验材料

TIB细胞保存液试剂盒[泰普生物科学（中国）有限公司]；主要成分为氯化钠、氯化钾、乙酸钙、甲醇、EDTA。

### 1.3 试验方法

#### 1.3.1 试验设计

本试验为回顾性研究，以依据临床症状和影像学阳性表现诊断为LM患者脑脊液作为研究对象，根据不同送检方式分为应用TIB细胞保存液试剂盒收集并送检脑脊液的标本（实验组）和常规应用腰穿包内无菌塑料试管收集并送检脑脊液的标本（对照组），将首次进行脑脊液细胞病理学检测患者的脑脊液纳入研究，探索不同固定送检方式对脑脊液细胞病理学阳性率的影响；应用*Logistic*回归分析结果评价脑脊液生化检测与脑脊液细胞病理学阳性率的相关性，并根据受试者工作特征曲线（receiver operating characteristic curve, ROC）评估脑脊液生化对肺癌LM的诊断价值；动态监测于我科接受培美曲塞鞘内化疗并进有完整的脑脊液细胞学及脑脊液生化（葡萄糖、总蛋白）检测≥2个周期的患者，研究培美曲塞鞘内注射治疗后脑脊液生化指标与细胞学变化对临床疗效的影响及对指导预后的临床应用价值。

#### 1.3.2 脑脊液采集方法

在无菌条件下对入组患者进行腰椎穿刺术，实验组为2021年1月-2021年11月共52例患者使用TIB细胞保存液试剂盒收集的5 mL脑脊液，对照组为2019年6月-2020年12月共53例患者使用腰穿包内无菌塑料试管收集的5 mL脑脊液，于2 h-4 h内送检病理科行脑脊液细胞病理学检查。将全部患者首次脑脊液送检的细胞病理学标本纳入脑脊液细胞病理学阳性率的研究。同时患者均使用2支无菌管各收集1 mL脑脊液分别于2 h-4 h内送检生化（总蛋白、葡萄糖）检测，留取标本后行培美曲塞鞘内注射治疗。

#### 1.3.3 治疗方法

培美曲塞鞘内注射前需要进行相应预处理，口服叶酸、肌注维生素B_12_，叶酸口服至末次鞘内用药结束后21 d；患者进行50 mg培美曲塞+1 mg地塞米松的鞘内注射化疗，每3周（21 d）为1个用药周期。

### 1.4 疗效评估

所有患者均经腰椎穿刺术留取脑脊液，记录脑脊液细胞学结果，生化项目指标（脑脊液葡萄糖正常值范围2.5 mmol/L-4.5 mmol/L、脑脊液总蛋白正常值范围180 mg/L-430 mg/L）。阳性结果判定为脑脊液见核异型或肿瘤细胞。治疗后根据RANO标准进行疗效评估。

### 1.5 统计分析

采用R语言统计软件进行数据统计与分析，所测结果满足正态分布且方差齐的变量采用*Student t*检验，方差不齐采用近似*t*检验，偏态分布的变量采用秩和检验，分类变量采用卡方或*Fisher*精确性检验。利用*Logistic*回归分析建立诊断模型，并通过ROC曲线评价诊断价值，*P* < 0.05为差异有统计学意义。

## 2 结果

### 2.1 一般临床资料结果

在2019年6月-2021年11月期间共入组105例患者，原发灶病理分型均为肺腺癌，其中男性31例，女性74例，平均年龄为53岁，所有患者表示出相应的大脑或脑膜受累症状，约90%患者存在相关基因突变，并均给予腰椎穿刺检查。详见表 1。

**表 1:** 脑膜转移癌患者基线特征 Clinical features of baseline patients with leptomeningeal metastases

Variables	Total	Experiment group (*n*=52)	Control group (*n*=53)	*P*
Age (yr)	53.81±8.18	53.84±8.59	53.79±7.76	0.889
Gender				0.481
Male	31 (29.52%)	17 (32.69%)	14 (26.42%)	
Female	74 (70.48%)	35 (67.31%)	39 (73.59%)	
Headache	81 (77.14%)	43 (82.69%)	38 (71.70%)	0.180
Sick and vomit	58 (55.24%)	36 (69.23%)	22 (41.51%)	0.004
Vision loss	42 (40.00%)	18 (34.62%)	24 (45.28%)	0.265
Hearing loss	36 (34.29%)	16 (30.77%)	20 (37.74%)	0.452
Movement disorder	53 (50.48%)	22 (42.31%)	31 (58.49%)	0.097
Confusion	13 (12.38%)	7 (13.46%)	6 (11.32%)	0.739
Previous chemotherapy	83 (79.05%)	37 (71.15%)	46 (86.79%)	0.049
Previous radiotherapy	53 (50.48%)	30 (57.69%)	23 (43.40%)	0.143
Targeted therapy	94 (89.52%)	43 (82.69%)	51 (96.23%)	0.024
*EGFR* exon 19 mutation	23 (21.91%)	6 (11.54%)	17 (32.08%)	0.011
*EGFR* exon 21 mutation	36 (34.29%)	18 (34.62%)	18 (33.96%)	0.944
*ALK* fusion mutation	16 (15.24%)	8 (15.39%)	8 (15.10%)	0.967
Only leptomeningeal metastases	52 (49.52%)	29 (55.77%)	23 (43.40%)	0.714
Combined with brain metastases	29 (27.62%)	15 (28.85%)	14 (26.42%)	0.395
Cerebrospinal fluid pressure (mmH_2_O)				
< 200	62 (59.05%)	32 (61.54%)	30(56.6%)	0.607
≥200	43 (40.95%)	20 (38.46%)	23(43.40%)	
EGFR: epidermal growth factor receptor; ALK: anaplastic lymphoma kinase.				

### 2.2 脑脊液细胞学检测结果

在无菌条件下对105例入组患者进行腰椎穿刺术行脑脊液细胞学检测，实验组中42例（80.77%）患者首次脑脊液细胞学检查结果为阳性，10例（19.23%）患者结果为阴性；对照组中24例（45.28%）患者首次脑脊液细胞学检查结果为阳性，29例（54.72%）患者首次检查结果为阴性，有统计学差异（*P* < 0.001）。

### 2.3 脑脊液生化检测辅助诊断结果

对105例患者均行脑脊液生化检查，其中于首次治疗前行脑脊液葡萄糖检查患者共105例，脑脊液葡萄糖 < 2.5 mmol/L共37例（35.24%），≥2.5 mmol/L共68例（64.76%），105例患者中脑脊液细胞学阳性共66例（62.85%），阴性共39例（37.14%）；首次治疗前行脑脊液总蛋白检查患者共71例，脑脊液总蛋白 < 430 mg/L共27例（38.03%），≥430 mg/L共44例（61.97%），71例患者中脑脊液细胞学阳性共53例（74.65%），阴性共18例（25.35%）。

首次行脑脊液生化检测的患者分别行*Logistic*回归分析结果显示，脑脊液葡萄糖 < 2.5 mmol/L发生脑脊液细胞病理学阳性风险是脑脊液葡萄糖≥2.5 mmol/L的2.456倍（OR=2.456, *P* < 0.05），脑脊液总蛋白≥430 mg/L发生脑脊液细胞病理学阳性风险是脑脊液总蛋白 < 430 mg/L的2.647倍（OR=2.647, *P* > 0.05）。ROC曲线显示，脑脊液中葡萄糖灵敏度为76.9%，特异度为54.5%，Youden指数为0.315，曲线下面积（area under the curve, AUC）为0.620；脑脊液中总蛋白灵敏度为44.4%，特异度为90.6%，Youden指数为0.350，AUC为0.671（图 1）。

**图 1:**
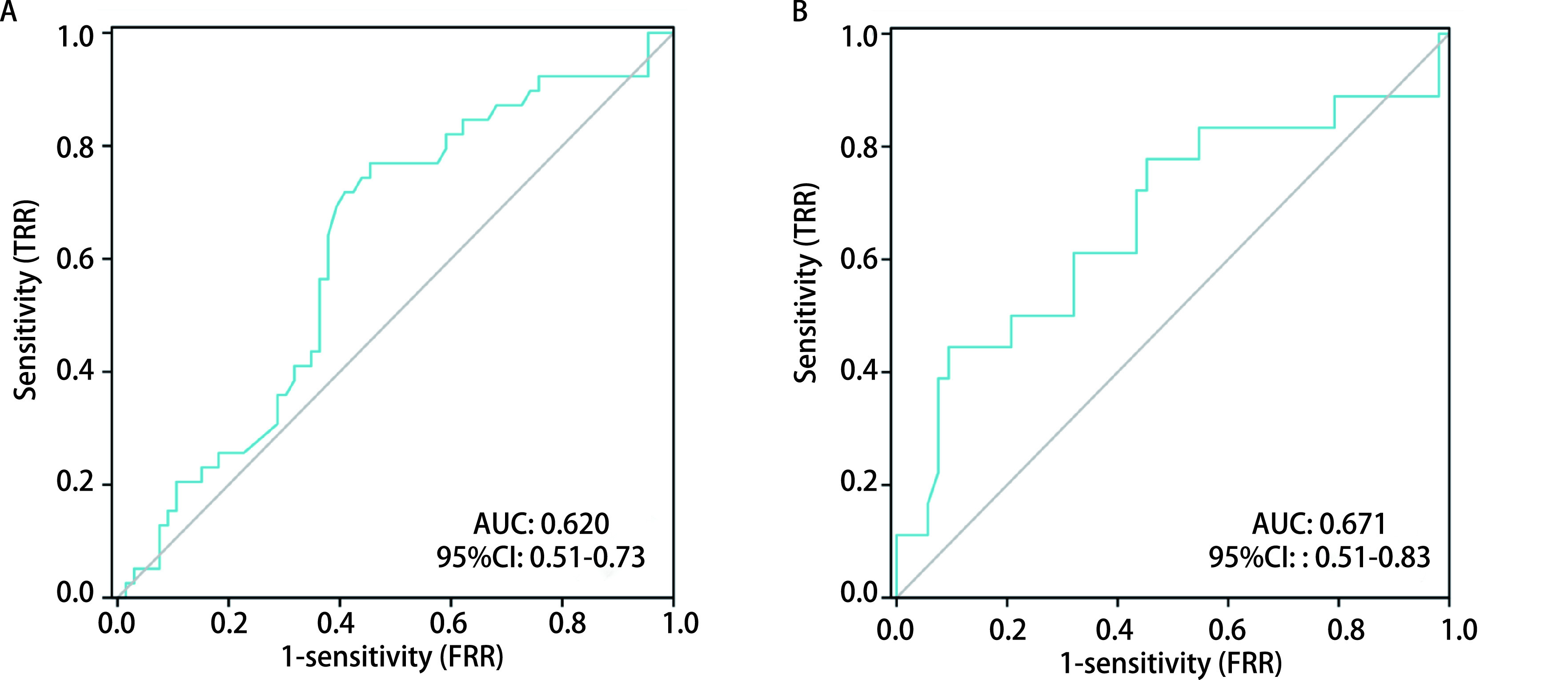
脑脊液生化检测的诊断价值。A：脑脊液葡萄糖诊断价值；B：脑脊液总蛋白诊断价值。 Cerebrospinal fluid biochemical testing's diagnostic utility. A: Diagnostic value of cerebrospinal fluid glucose test; B: Diagnostic value of cerebrospinal fluid total protein; AUC: area under the curve.

### 2.4 培美曲塞鞘内治疗后脑脊液生化检测结果

#### 2.4.1 脑脊液葡萄糖检测结果

本部分共纳入73例行2个周期培美曲塞鞘内注射治疗后，有完整的脑脊液细胞学和脑脊液生化（葡萄糖）检测的患者，脑脊液细胞学转阴率逐渐增加，治疗1个周期后转阴率为4.35%，治疗2个周期后为13.04%。治疗2个周期后脑脊液葡萄糖水平较治疗前升高，有统计学差异（*P* < 0.001）（表 2）。对首次脑脊液细胞学中46例阳性患者，27例阴性患者治疗后脑脊液葡萄糖分别做横向、纵向评估，治疗2个周期后葡萄糖水平均升高，无统计学差异（*P* > 0.05）（表 3）。

**表 2:** 治疗前后脑脊液生化变化情况 Cerebrospinal fluid biochemical changes before and after treatment

Course of treatment	Cerebrospinal fluid glucose level (mmol/L)	Cerebrospinal fluid total protein level (mg/L)
Before the treatment	3.120 (2.590, 3.670)	528 (295, 894)
1 wk after the treatment	3.020 (2.300, 3.410)	512 (346, 1,001)
2 wk after the treatment	3.290 (3.020, 3.620)^a^	532 (362, 848)
Compared with before the treatment, ^a^*P* < 0.001.		

**表 3:** 首次脑脊液细胞学阴性、阳性组患者治疗前后脑脊液葡萄糖变化情况 Changes of CSF glucose in the cytological negative and positive groups before and after treatment

Variables	Positive group (*n*=46)	Negative group (*n*=27)	*Z*	*P*
Before the treatment	2.8 (2.28, 3.26)	3.080 (2.870, 3.550)	-1.914	0.056
1 wk after the treatment	3 (2.490, 3.720)	3.130 (2.720, 3.610)	-0.434	0.668
2 wk after the treatment	3.28 (2.810, 3.610)	3.410 (3.030, 3.780)	-0.817	0.417
CSF: cerebrospinal fluid.				

#### 2.4.2 脑脊液总蛋白检测结果

50例行2个周期培美曲塞鞘内注射治疗后，并有完整的脑脊液细胞学和脑脊液生化（总蛋白）检测患者中，脑脊液细胞学转阴率逐渐增加，治疗1个周期后转阴率为5.26%，治疗2个周期后为13.16%。治疗2个周期后脑脊液总蛋白水平较治疗前升高，差异无统计学意义（*P* > 0.05）（表 2）。对首次脑脊液细胞学中38例阳性患者、12例阴性患者治疗后脑脊液总蛋白分别做横向、纵向评估，首次细胞学阳性患者中治疗2个周期后脑脊液总蛋白水平降低，阴性患者中脑脊液总蛋白水平升高，无统计学差异（*P* > 0.05）（表 4）。

**表 4:** 首次脑脊液细胞学阴性、阳性组患者治疗前后脑脊液总蛋白变化情况 Changes of total protein in CSF cytology negative and positive groups before and after treatment

Variables	Positive group (*n*=38)	Negative group (*n*=12)	*Z*	*P*
Before the treatment	659 (388, 1,044)	427 (245, 538)	1.715	0.088
1 wk after the treatment	596 (338, 909)	489 (264, 567)	1.318	0.192
2 wk after the treatment	553 (390, 854)	494 (288, 658)	0.999	0.323

## 3 讨论

脑脊液作为LM的最佳液体诊断标本，是目前细胞病理学诊断的主要载体，提高LM脑脊液中细胞病理学诊断的阳性率一直是临床研究的热点。

LM患者早期表现并不典型，晚期由于脑脊液回流受阻，会出现相应的颅内压增高表现。既往关于实体瘤或淋巴瘤的LM研究中，头痛、精神状态异常和颅神经功能障碍是最常见的症状^[10]^。磁共振成像（magnetic resonance imaging, MRI）上的特征性发现是脑膜强化，最明显的是在小脑叶之间的颅底，沿颅神经以及脊髓和神经根周围，在对187例LM患者的回顾性研究中显示，53%的患者通过影像学确诊，23%通过细胞学确诊，24%通过两者共同确诊，但值得关注的是，LM影像学一经确诊提示预后极差^[11, 12]^。由于不显著的影像学表现和早期临床症状，使LM误诊或漏诊概率增加，从而丧失最佳治疗时间，因此脑脊液细胞学结果就尤为重要。脑脊液被认为是*EGFR*突变型NSCLC中LM最有代表性的检测标本^[13, 14]^，但是研究表明LM患者在首次脑脊液样本中发现肿瘤细胞的概率不超过50%^[4]^。实际的临床工作中，由于脑脊液细胞学阳性率不高，导致部分LM患者诊断不明确，并且由于LM患者疾病进展快、预后差的特点，不及时的诊断使我们的治疗也变得相对困难。

本研究在前期临床工作中发现，经影像学及临床症状明确诊断为LM的患者中，脑脊液细胞学阳性率不显著，是否脑脊液细胞学检查的敏感性在一定程度上受到了脑脊液细胞学检查方式的影响，因此我们提出如何能在临床工作中简便、高效地提高脑脊液细胞学阳性率。在本试验结果中，首次脑脊液细胞学阳性率（80.77%）远高于既往研究，我们分析实验组中TIB细胞保存液试剂盒的成分主要为氯化钠、氯化钾、乙酸钙、甲醇、EDTA等，其中氯化钠、氯化钾在细胞固定时使细胞内外维持一个稳定、合理的渗透压，甲醇具有缩短固定时间、结构显示清晰等优点^[15]^。综合相关因素考虑脑脊液的离体固定可认为是临床工作中重要的组成部分，也是核心元素之一，在对脑脊液进行离体后立即固定的方式，可能使脑脊液中的肿瘤细胞在外界环境中变化的概率下降，有助于细胞形态和结构的完整表达，并且此方式简便、高效，可作为临床工作中提高脑脊液细胞学阳性检出率的有效方法。

由于细胞学分析的敏感性尚不完善，因此还在寻找是否存在相关检测指标的异常变化，可以在临床工作中高度警示LM发生，降低漏诊率。有研究^[16, 17]^显示由于脑脊液流动受阻，至少50%的患者的脑脊液压力升高，约80%的病例脑脊液蛋白浓度升高，在没有感染的情况下，25%-40%的病例葡萄糖水平降低。脑脊液检查结果的异常可提示（但非诊断性）LM的发生。理想情况下，这些指标不仅具有诊断价值，而且具有预后价值，从而有助于选择适当的治疗方法。本试验通过对脑脊液生化检测*Logistic*回归分析结果得出，脑脊液葡萄糖 < 2.5 mmol/L是LM具有诊断价值的因素（*P* < 0.05）。在ROC曲线中，AUC数值越接近于1，表示诊断效果越佳，通过分析ROC曲线得出两者AUC值均提示对LM患者具有一定的诊断价值。通过此研究提示在临床工作中，当肿瘤患者存在阳性临床症状且脑脊液葡萄糖降低、总蛋白升高时应高度怀疑LM的可能。并且对于具有LM临床症状的患者，如果出现脑脊液细胞学诊断阴性应该进行连续动态检测并伴随理化检查及相关基因检测（驱动基因阳性肺癌患者），可以提高LM诊断的阳性率。

患者生活质量的提高和生存期的延长仍是LM的诊治目标。既往研究^[18]^中已确定的影响预后的因素包括：低肿瘤患者卡氏（KarnofskyPerformance Status, KPS）评分、多发的中枢神经系统疾病、严重的神经功能障碍、脑实质病变等。有研究^[19]^显示脑脊液中大量循环肿瘤细胞消耗葡萄糖，脑膜的广泛和弥漫性受累可能会导致葡萄糖转移受损，导致脑脊液中葡萄糖减少，并且脑脊液中葡萄糖减少、蛋白升高是LM的患者中不良预后因素。但是Hitchins等^[20]^报告中初始脑脊液蛋白升高（> 0.5 g/L）的患者却比初始水平较低的患者临床缓解率更高（65% *vs* 13%, *P* < 0.05）。因此相关指标的临床意义应继续探讨。

鞘内化疗的优点在于可以不通过血-脑屏障障碍，通过腰椎穿刺等给药手段将药物直接注入脑脊液中，小剂量获得高浓度^[21]^。培美曲塞联合铂类目前被认为是晚期NSCLC，特别是非鳞状细胞恶性肿瘤患者的一线治疗方案^[22, 23]^。在我科前期研究^[5]^中，确定了培美曲塞50 mg为NSCLC LM患者鞘内注射的安全剂量，并且培美曲塞鞘内化疗具有良好的临床疗效和安全性。本试验通过对同时于我科接受培美曲塞鞘内化疗≥2个周期进行动态监测发现，在脑脊液细胞学结果中，治疗后脑脊液细胞学转阴率均逐渐增加，提示鞘内治疗对肿瘤细胞的清除存在相关作用。脑脊液生化检测方面，脑脊液葡萄糖水平治疗2个周期后较治疗前升高，存在统计学差异（*P* < 0.001）。由此我们考虑是否可以将脑脊液葡萄糖作为指导预后的指标之一，继续进行多周期鞘内注射治疗患者的研究，通过动态监测脑脊液中葡萄糖变化，综合多因素评估治疗效能，通过脑脊液葡萄糖的变化寻找合适的鞘内治疗周期及持续时间等。本试验的不足在于，作为回顾性研究，受到患者经济条件、脑脊液取样的标本量及自身条件限制，未能对同一患者进行自身对照的前瞻性研究。理论上来讲总蛋白含量可能成为阳性诊断的指标，但是本试验结果不明显，考虑脑脊液生化检测未采用细胞固定液进行检测，因此后续试验中建议所有脑脊液样本送检生化均采用Streck Cell-Free DNA BCT试管送检，并在扩大样本量的基础上行后续探讨。

综上所述，脑脊液离体后的立即固定处理并送检是影响LM患者脑脊液细胞学阳性检出率的重要因素，这种看似简单的操作可以显著影响LM的诊断率，应该在临床工作中给予充分的重视。对于临床症状和影像学阳性初步诊断为LM的患者，采取细胞病理学和脑脊液生化指标综合评估可以提高LM的检出率，并且当肺癌患者出现脑膜刺激症状时，应给予高度重视，早诊断早治疗会延长患者的生存时间、提高患者的生存质量。
